# Development and evaluation of a decision-making aid for couples hesitant about transitioning from infertility treatment to advanced assisted reproductive technology: a usability and feasibility study

**DOI:** 10.1186/s13104-023-06652-0

**Published:** 2023-12-08

**Authors:** Kyoko Asazawa, Kaori Takahata, Natsuko Kojima, Hiromi Onizawa, Masami Kawanami, Atsumi Yoshida, Kumiko Hasegawa, Makoto Chihara, Naoko Arimori

**Affiliations:** 1 Division of Nursing, Tokyo Healthcare University, 2-5-1 Higashigaoka, Meguro, Tokyo, 152-8558 Japan; 2 Division of Nursing, Shonan Kamakura University of Medical Sciences, Kanagawa, Japan; 3 The Reproduction Center, Kiba Park Clinic, Tokyo, Japan; 4 Mia Grace Clinic Niigata, Niigata, Japan; 5 Graduate School of Health Sciences, Niigata University, Niigata, Japan

**Keywords:** Infertility, Decision making, Feasibility studies

## Abstract

**Objectives:**

The aims of this study were (1) to develop a decision-making aid for couples hesitant about transitioning from infertility treatment to advanced assisted reproductive technology, (2) to examine the adequacy of this aid, and (3) to evaluate its usability. After the first version of the decision-making aid was created, the first version was supervised and finally a prototype of the decision-making aid was completed. We conducted a feasibility study from February to March 2022. We used a quantitative cross-sectional descriptive design involving 22 medical professionals and infertility survivors recruited.

**Results:**

Twenty-two participants (3 reproductive medical specialists, 11 nurses who specialize in reproductive medicine, and 8 infertility survivors) were included in the final analysis (91.7% valid response rate). Of these participants, 81.8% answered *Agree* regarding “**Easy-to-read degree of charts**”, 17 (77.3%) answered *It is just the right amount* regarding “**Appropriateness of information volume**”, 81.8% answered *Agree* regarding “**Ease of understanding content**”, and 90.9% answered *Good* regarding “**Overall performance**”. From the opinions received, we extracted 4 categories: “Useful for decision making,” “Suitable for providing information,” “Useful in clinical practice,” and “Needs improvement.” Certain degrees of surface validity and content validity were confirmed for the trial version of the decision-making aid.

## Introduction

The number of women suffering from infertility worldwide is estimated at 40.5–186 million, and this number has continued to grow over the years [1, 2]. Conversely, the number of newborns conceived using advanced assisted reproductive technology (ART) has also increased year by year [3–5]. Patients undergoing infertility treatment have physical, psychological, economic, and time burdens [6]. In particular, various problems have been experienced by patients undergoing ART, such as increased frequency of multiple pregnancies [7], age-related problems [8], non-insurance coverage of the expensive medical treatment [8], increased depression and anxiety during the ART cycle [9], and psychological distress [9, 10]. Stress and fear due to ART failure have also been experienced [11]. Specifically, the high cost and burden of ART cause stress among care recipients [12], making it difficult for them to transition from infertility treatment to ART.

During infertility treatment, women experience great conflict in determining the optimal stages of treatment, including the correct timing and methods such as artificial insemination [13]. Acceptance of ART is influenced by a couple’s attitude, family’s mindset, and their perceptions [14]. Addressing decision-making conflicts in couples undergoing fertility treatment is an important undertaking of healthcare providers [15]. In Japan where there is no age limit for fertility treatment, there are still no standards for terminating such treatment. This results in a conflict between continuing and terminating fertility treatment [16]. Therefore, there is a need for a decision-making support for resolving decision-making conflicts in transitioning from fertility treatment, including information about treatment termination. A decision-making aid serves as a supplementary tool when presented with two or more options. A systematic review [17] that looked into the effects of a decision-making aid showed increased knowledge, less conflict, and greater satisfaction with the couple’s individual decisions. Decision-making aids, such as those from the Ottawa Hospital Research Institute [18], usually consist of a collection of aids for various themes. However, to our knowledge, there is still no decision-making aid for transitioning from infertility treatment to ART. The aims of this study were (1) to develop a decision-making aid for couples hesitant about transitioning from infertility treatment to ART, (2) to examine the adequacy of this decision-making aid, and (3) to evaluate its usability.

## Methods

### Participants and procedures

Cooperation for research participation was obtained from the directors of two infertility treatment facilities. The inclusion criteria were as follows: (1) doctors specializing in reproductive medicine who are involved in fertility treatment, (2) nurses/midwives qualified as reproductive consultants or certified nurses involved in fertility nursing, and (3) ART-experienced patients. This distribution made it possible to obtain representative opinions based on treatment experience. The exclusion criterion was those in whom written consent was not obtained. The sample size was estimated as 22 participants following the calculation procedure of Nielsen & Landauer [19]. Assuming a sample size of λ = 0.20 needed to detect 85% of problems regarding usability of treatment, and an expected dropout rate of 10%, we calculated that 22 people would be needed for the study. In this regard, we planned to recruit 6 to 7 each of medical doctors, nurses/midwives, and previous patients.

The survey period was from February 2023 to April 2023. The participants were introduced by the director of the research cooperating facility. The researchers explained the aims of the study to the participants verbally and in writing. After giving consenting, the participants were given a booklet on “Decision-Making Aids” and a questionnaire. After thoroughly viewing the decision-making aid prototype, the participants completed an anonymous questionnaire and mailed it individually.

### Development of a decision-making aid

#### 1) Preparation of the first version of the decision-making aid

We conducted a literature survey on the contents and methods of support for making decisions in patients undergoing fertility treatment. Then, we selected the contents to be included in the decision-making aid. All contents from published papers included in the decision-making aid were approved by Japanese academic societies and authors [20–25]. The decision-making aid was developed to help patients undergoing infertility treatment be able to make decisions when they are hesitant about “transitioning from general infertility treatment to ART” and “ending treatment”. The original decision-making aid was created based on the decision aids from Ottawa Hospital Research Institute [18]. The decision-making aid was specifically developed following the decision-making guide of the *International Patient Decision Aid Standards instrument* (IPDASi) [26] to meet all the qualification, accreditation, and quality standards.

#### 2) Supervision of the first edition of decision-making aid

The development of the first edition of the decision-making aid was extensively supervised by several reproductive medicine specialists and clinical geneticists. The contents were revised each time.

#### 3) Completion of decision-making aid prototype

The applicability of the decision-making aid in clinical practice was supervised by reproductive medicine specialists and reproductive counsellors at infertility centers.

Content corrections were made based on the comments received, and a temporary version of the decision-making aid was completed in a booklet format. The decision-making aid consisted of No. 1 to No. 4 items. **No. 1 was about “What is the method for making a decision?**”, and included various aspects such as “confirmation of who can use it”, “how to select fertility treatment”, and “how to use the decision-making aid”. **No. 2 consisted of** “**What are your options?**”, and included various aspects such as “considering infertility treatment methods”, “comparison of options (general infertility treatment or ART)”, and “suspension/interruption of treatment”. **No. 3 focused on “Think about your option**”, to consider how important the contents are such as “receiving infertility treatment”, “having a child”, and “relationship with a partner”. **No. 4 described the** “**Decide**”, such as “confirm your feelings” and “respond if you cannot decide”. To confirm the participants’ feelings, we used the SURE test [27–30].

### Assessment instruments

#### 1. Demographics

We asked the participants about their gender, occupation, years of work experience, years of treatment experience (previous patients only), and preferred selection method.

#### 2. Evaluation of the face validity of the trial version of the decision-making aid

We asked for a single response by setting options for viewing time, degree of browsing, appropriateness of size, appropriateness of information volume, easy-to-read degree of charts, appropriateness of number of pages, and types of media that are easy to use.

#### 3. Assessing the surface validity of the trial version of the decision-making aid

We set options for ease of understanding, usability, degree of recommendation to patients, balance of content, and overall impression, and asked for a single response.

#### 4. Opinions regarding the trial version of the decision-making aid from the participants

The participants were asked to freely describe their opinions on what they liked about the trial version of the decision-making aid, what they did not like, what other contents were necessary, and what they needed to improve the decision-making aid.

### Data analysis

Basic statistics for each variable were calculated, and the face validity and content validity of the decision-making aid were obtained from the frequency distribution tables. After summarizing the free-text data, we divided the data into labels and extracted categories for each similar content. The open-ended remarks were analyzed using constant comparative analysis.

## Results

**Fig. 1:**
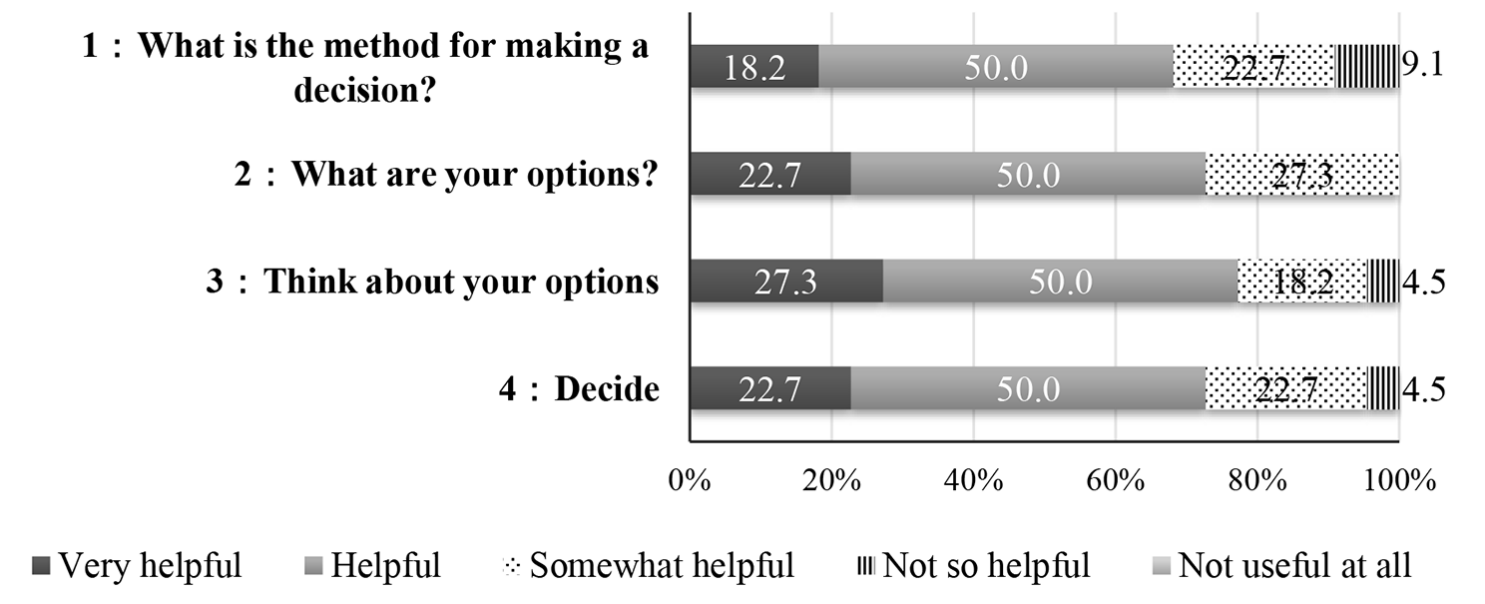
Evaluation of the usefulness of each content of the decision-making aid (N = 22)

Valid responses were obtained from a total of 22 participants, which included 3 reproductive medical specialists, 11 nurses who specialize in reproductive medicine, and 8 infertility survivors (valid response rate, 91.7%). The evaluation of the participants for each content of the decision-making aid prototype were as follows: **1: What is the method for making a decision?** These responses were *Very helpful* (18.2%), *Helpful* (50.0%), and *Somewhat helpful* (22.7%); **2: What are your options?** These responses were *Very helpful* (22.7%), *Helpful* (50.0%), and *Somewhat helpful* (27.3%); **3: Think about your options?** These responses were *Very helpful* (27.3%), *Helpful* (50.0%), and *Somewhat helpful* (18.2%); **4: Decide.** These responses were *Very helpful* (22.7.%), *Helpful* (50.0%), and *Somewhat helpful* (22.7.%) (Fig. 1).

The average viewing time was 22.8 min, and 77.3% of the participants answered *It is just the right amount* regarding **Appropriateness of information volume** (Table 1). Appropriateness of the surface was rated on a scale of 5, with 81.8% evaluating the charts as easy to read. Regarding the Appropriateness of number of pages, 45.5% of the participants answered *just the right amount*, and 54.5% answered *slightly more.* Regarding Ease of understanding content, 81.8% of the participants answered *Agree*. Regarding Usefulness when selecting ART, 90.9% answered *Agree*. Regarding Availability at the end of treatment, 90.9% answered *Agree*. Regarding Degree of recommendation to patients, 81.8% answered *Agree*. As regards Overall performance, 81.8% of the participants responded that the overall performance was *Very good*, 81.8% as *Good*, and 9.1% as *Normal.*

**Table 1:** Face validity content validity evaluation of decision-making aid trial version (N = 22)

items		Mean	±SD
		n	%
Viewing time (minutes)		22.8	± 12.4
Range of viewing time		(7~60)	
Appropriateness of information volume	too much	4	18.2
	the amount is too small	1	4.5
	It’s just the right amount	17	77.3
Easy-to-read degree of charts	Strongly agree	2	9.1
	Agree	16	72.7
	Neither	2	9.1
	Disagree	2	9.1
	Strongly disagree	0	0.0
Appropriateness of number of pages	too many	0	0.0
	slightly more	12	54.5
	just the right amount	10	45.5
	slightly less	0	0.0
	too few	0	0.0
Ease of understanding content	Strongly agree	5	22.7
	Agree	13	59.1
	Neither	4	18.2
	Disagree	0	0.0
	Strongly disagree	0	0.0
Usefulness when selecting ART	Agree	20	90.9
	Disagree	0	0.0
	I don’t know	2	9.1
Availability at the end of treatment	Agree	20	90.9
	Disagree	0	0.0
	I don’t know	2	9.1
Degree of recommendation to patients	Agree	18	81.8
	Disagree	0	0.0
	I don’t know	1	4.5
	No response	3	13.6
Balance of contents			
Information is biased towards specific options		3	13.6
Information is well-balanced		19	86.4
Overall performance	Very good	2	9.1
	Good	18	81.8
	Normal	2	9.1
	Bad	0	0.0
	Very bad	0	0.0

ART, assisted reproductive technology

The content analysis of the open-ended responses revealed 4 categories: (1) **Useful for decision making**, (2) **Suitable for information provision**, (3) **Useful in clinical practice**, and (4) **Needs improvement** (Table 2).

**Table 2:** Opinions regarding the trial version of the decision-making aid from the participants (N = 22)

Category code	Code
Useful for decision making	Serve as a guide for treatment selection
	Able to confirm values for treatment and children
	Useful for organizing the patient’s thoughts
Suitable for information provision	Easy to understand specific numbers and data
	Patients can understand how to use
	Useful for sharing information with partners
	Allows quantitative comparison between artificial insemination and ART
Useful in clinical practice	Easy for medical staff to use when explaining
	Useful as materials that can be handed over in busy worksites
	Medical workers also learn
Needs improvement	Some citations are outdated and need to be changed
	Consideration is required due to the large amount of text
	Additions to testimonials and model cases are required

ART, assisted reproductive technology

## Discussion

In this study, we developed a decision-making aid for couples hesitant about transitioning from infertility treatment to ART, examined the adequacy of the decision-making aid, and evaluated its usability. The face validity and content validity of the decision-making aid showed high practical usefulness and high comprehensibility. From the overall results, the decision-making aid was assessed to be useful and applicable to patients undergoing infertility treatment.

Notably, there were also some contents that were not very helpful, and the participants did not completely agree with these contents. In the **Ease of understanding content**, 18.2% of the participants *Neither* agreed nor disagreed with the understandability of the content, and 9.1% of the participants did not find the decision-making aid **useful when selecting ART** or ending treatment.

Thus, there is a need to scrutinize more thoroughly such information on the decision-making aid. Additionally, some of the quotes were outdated and needed updating. As fertility treatment is constantly evolving, treatment-related content must be revised with the utmost care. Putting things into perspective, the decision-making aid was originally made not only to provide information, but also to help those undergoing infertility treatment to make decisions on their own with conviction. In the evaluation of the usefulness of each content of the decision-making aid (**Fig. 1**), particularly the third category “**Think about your options**”, the patients can think about how important it is to “get fertility treatment”, “get ART”, and “have a child”.

A decision-making aid is used for complex health decisions [31]. Such aid has one or more rational options, each with its own strengths and weaknesses, and it is up to the values of an individual to determine which option is best [26]. A decision-making aid is intended to inform patients of their options, clarify their personal values, and facilitate discussions with their healthcare providers [26]. Such aid improves knowledge, the accuracy of risk perception, alignment with personal values, and conflict [17]. This facilitates decision-making, coupled with greater satisfaction and less decision regret [32]. A decision-making aid is suggested to reduce treatment conflict and enables more informed decision-making in patients undergoing infertility treatment. In the future, it is necessary to use a decision-making aid in actual patients to evaluate its usefulness.

## Conclusions

We developed a decision-making aid for couples hesitant about transitioning from infertility treatment to ART. We also examined its adequacy evaluated its usability. More than 81.8% of reproductive physicians, reproductive nurses, and those who have experienced infertility treatment rated the content validity of the decision-making aid highly. The participants opined that the decision-making aid was “Useful for decision making,” “Ideal for providing information,” and “Useful in clinical practice,” but it also “Needs improvement”. Couples hesitant about transitioning from infertility treatment to ART or ending infertility treatment can obtain correct evidence-based information, which is anticipated to be helpful for self-decision. Midwives and nurses can also use the decision-making aid to provide evidence-based information when supporting the decision-making of couples who have doubts on using ART or ending their infertility treatment.

### Limitations

The decision-making aid described herein is in the post-development stage and is being evaluated by healthcare professionals and previous fertility patients. Patients undergoing infertility treatment have not yet been evaluated. The corrections pointed out by such patients have not yet been incorporated in the decision-making aid.

## Data Availability

All data generated or analyzed during this study are included in this article.

## Abbreviations

ART: Assisted Reproductive Technology
